# Dynamic calibration with approximate Bayesian computation for a microsimulation of disease spread

**DOI:** 10.1038/s41598-023-35580-z

**Published:** 2023-05-27

**Authors:** Molly Asher, Nik Lomax, Karyn Morrissey, Fiona Spooner, Nick Malleson

**Affiliations:** 1 School of Earth and Environment, University of Leeds, Leeds, LS2 9JT UK; 2 School of Geography, University of Leeds, Leeds, LS2 9JT UK; 3 British Library, Alan Turing Institute, London, NW1 2DB UK; 4 Department of Management, DTU Technical University of Denmark, Copenhagen, Denmark; 5 Our World in Data, Global Change Data Lab, Oxford, UK

**Keywords:** Infectious diseases, Computational science, Software, Psychology and behaviour

## Abstract

The global COVID-19 pandemic brought considerable public and policy attention to the field of infectious disease modelling. A major hurdle that modellers must overcome, particularly when models are used to develop policy, is quantifying the uncertainty in a model’s predictions. By including the most recent available data in a model, the quality of its predictions can be improved and uncertainties reduced. This paper adapts an existing, large-scale, individual-based COVID-19 model to explore the benefits of updating the model in pseudo-real time. We use Approximate Bayesian Computation (ABC) to dynamically recalibrate the model’s parameter values as new data emerge. ABC offers advantages over alternative calibration methods by providing information about the uncertainty associated with particular parameter values and the resulting COVID-19 predictions through posterior distributions. Analysing such distributions is crucial in fully understanding a model and its outputs. We find that forecasts of future disease infection rates are improved substantially by incorporating up-to-date observations and that the uncertainty in forecasts drops considerably in later simulation windows (as the model is provided with additional data). This is an important outcome because the uncertainty in model predictions is often overlooked when models are used in policy.

## Introduction

The global COVID-19 pandemic brought considerable public and policy attention to the already rich field of infectious disease modelling^1^. In the UK, the predictions of the ‘CovidSim’ individual-based model^2^ were the foundation for many government policies, whilst globally policymakers and the public placed greater trust in modelling than ever before^1^.

In providing policymakers with robust data and scenarios, a major hurdle that modellers must overcome is model uncertainty. Uncertainties arise from a variety of different sources, including parameter uncertainty (imperfect knowledge of the model parameters), model structure uncertainty (uncertainty about the fundamental model structure), and scenario uncertainty (uncertainty about the specific scenario to which the model will be applied)^3^. For example, estimates of the reproduction number ($R_0$) varied considerably during the pandemic and the different choices of $R_0$ is one of the reasons why for example the ‘Oxford’^4^ and ‘Imperial’^2^ models in the UK, had very different predictions^5^.

Although some sources of uncertainty are difficult or even impossible to constrain (i.e., scenario prediction), other sources can be reduced as more data and information about the spread of the disease becomes available. Indeed, as many nation states began to implement wide-ranging COVID-19 testing regimes^6^, these data, often updated on a daily basis, were used to re-calibrate models in (near) real-time to reduce parameter uncertainty. The use of real-world observations to improve model predictions is not new. *Data assimilation*—a suite of mathematical techniques that can be used to update the state of a model with new information—is one of the reasons why weather forecasts have improved so dramatically in recent decades^7^. However, the application of data assimilation techniques to individual-based models, as required to model the spread of infectious diseases, is extremely difficult and has only been attempted on the simplest systems^8–12^.

The aim of this paper is to adapt an existing, large-scale, individual-based COVID-19 model, the DyME model^13^, to explore the benefits of updating the model in pseudo-real time as new case data emerge using Approximate Bayesian Computation (ABC) techniques. As full data assimilation for a disease model with thousands or millions of individuals is not yet possible, this paper uses ABC to dynamically recalibrate the model’s *parameter values* but not the *model state*. ABC offers advantages over one-shot calibration techniques—i.e. those that provide a single, ‘optimal’ estimate of the model’s parameters—by providing information about the uncertainty associated with particular parameter values and the resulting COVID-19 predictions. This not only provides information about how uncertain the model’s predictions are, but can also show how parameter values (and their uncertainty) evolve over the course of the pandemic as changes in human behaviour influence the spread of the disease. Looking at such distributions is crucial in fully understanding a model and its outputs, as retrospective analysis of the UK’s ‘CovidSim’ model showed^3^.

Specifically, the aims of this paper are to:

1. Evaluate the extent to which the dynamic re-calibration of a large-scale, individual-based COVID-19 model improves the accuracy of the model’s predictions;
2. Explore whether the change in parameter values, and their uncertainties, reveal useful information about the propagation of the disease throughout the pandemic.

By incorporating up-to-date observations, we find that forecasts of future disease infection rates are improved and that the uncertainty in forecasts drops considerably in later simulation windows. With the addition of new data in the later stages of the pandemic, the model becomes much more certain about the future evolution of the disease. This is an important outcome as the uncertainty in model predictions is often overlooked when models are used in policy^14^. Although dynamic re-calibration is shown to improve model predictions, the overall aim of this programme of work is to ultimately implement data assimilation algorithms that are able to update the model state as well as its parameters in light of new data. This approach may be more successful because it would allow manipulation of the state of individual model instances. However, the field needs much more work in adapting data assimilation methods for large, complex individual-level models first, as currently the only published examples of the use of data assimilation in the context of individual-level modelling are on relatively simple systems.

## Background

### Compartmental models of disease spread

Mathematical/epidemiological models can be used to help understand the spread, and therefore guide prevention, of infectious diseases. Epidemiological models can be broadly split into two categories: compartmental models and individual-based models (including agent-based models). Of the two approaches, compartmental models are the most commonly used. Compartmental models are a form of equation-based modelling and are often based on the Susceptible-Infected-Removed (SIR) approach first formulated by Kermack^15^, or the extended Susceptible-Exposed-Infected-Removed (SEIR) model introduced by Rvache^16^. In both cases, the population is split between different compartments each representing different disease states. The evolution of the system, and the proportion of the population within each compartment, is governed by a set of differential equations.

Compartmental models simply and effectively capture the coarse scale dynamics of the spread of infectious diseases at a low computational cost^17,18^. Their relative mathematical simplicity also lends itself well to data assimilation frameworks. However, regarding the representation of actual population dynamics, these models suffer from several drawbacks including the simplified assumption that the population is equally mixed, and that all population members come into contact with each other with equal probability. This conflicts with evidence of human social dynamics which suggests that the majority of human contacts actually occur within limited social networks^19^. Consequently, compartmental models are unable to account for exact patterns of contact which, especially at the disease outbreak when infections are low, are crucial in determining the path of disease transmission^20^. It is also assumed that all individuals in the model behave in a similar way. The aggregate effect of the actions of all individuals within one compartment, rather than individual behaviours, is modelled^21^. This precludes the use of compartmental models to study questions related to individual-level behaviours and the evolution of such behaviour in response to external stimuli such as policy recommendations. Compartmental models also struggle to adjust to the evolution of diseases and epidemics, for instance the arrival of new variants or vaccine versions, as modelling these requires the addition of separate compartments to the model^22^.

### Individual-based models (IBMs)

Representing individuals and their characteristics explicitly, ‘individual-based model’ (IBM) refers to modelling techniques such as dynamic microsimulation^23^ and agent-based modelling^24^. Unlike compartmental models, IBMs assign discrete individuals their own characteristics and behaviours and each individual is simulated directly. In infectious disease modelling, each individual has an infection state and interacts with other individuals according to decisions based upon probabilistic rules. These contacts result in the transmission of the disease from individual to individual. The aforementioned ‘CovidSim’^2,25^, which was one of the key drivers of UK’s response to COVID-19, is an IBM.

Whilst IBMs simulate the dynamics of a disease in a human population in a more realistic way than compartmental models, there are a number of well-known challenges that they must overcome^26,27^. Of these, difficulties in the calibration (finding appropriate parameter values) and validation (ensuring that the model adequately replicates real-world data) are the most pressing in the context of disease modelling. Ideally, IBMs should be evaluated at multiple (including individual-level) scales^28^. However, calibration and validation are difficult with IBMs because: (i) the models typically simulate (and are themselves) complex systems so exhibit nonlinear relationships between parameters, prohibiting linear optimisation approaches; (ii) the high degree of stochasticity in individual disease transmission makes IBMs highly uncertain, so multiple model runs with identical parameter values can lead to different outcomes; (iii) IBMs are often very computationally expensive, so running the large numbers of models required to robustly calibrate a model can be prohibitively expensive. In addition, although the models themselves are extremely rich, the data used to calibrate/validate them are often relatively coarse. For example, while COVID-19 IBMs represented the different stages of disease transmission at the individual level in great detail, the only data on which to evaluate the models, such as positive tests or deaths, were aggregated and sparse, and as such only quantified a tiny part of the underlying dynamics of disease spread.

In the absence of high-resolution data at multiple scales, modellers must make the best use of the data that are available. One means of achieving this is to regularly update a model as new (albeit coarse) data become available to ensure that model predictions are based on the most up-to-date information.

### Dynamic updating of disease models

A general problem that disease models must face is that the underlying systems are extremely complex, so constraining a simulation to the ongoing evolution of the real system is challenging. Even a perfectly calibrated disease model will diverge from the real system over time.

Data assimilation is a technique developed originally for numerical weather prediction^7^. It attempts to overcome the difficulty of predicting the behaviour of chaotic systems in which small changes in initial conditions can propagate into large changes in system outputs. It does this by combining imperfect models with imperfect observations through a successive time-stepping process. At each time-step, the model predictions are compared with observations, and corrections applied, before the model continues to run for another time-step.

Previous research has applied data assimilation to compartmental epidemiological models of infectious diseases including Cholera, Ebola, Influenza and, most recently, COVID-19. For instance, data assimilation has been used in combination with SEIR models^29–32^; with an SIR model^33^ and with an SIRS model^34^. These applications have primarily used a Kalman filter^32,35^, a particle filter^29,30^, or both^31,34^. The application of data assimilation to individual-level disease models is, however, much rarer, with only two relevant examples available at the time of writing. The first^30^, applied a modified particle filter to an agent-based model (ABM) that simulates the spread of Ebola in west Africa. Here the ABM is based on a standard SEIR model, modified so that the transition of individuals through the states is defined by a social contact network. ‘Beam’ particle filtering is used to estimate a posterior distribution of the parameters that control the model disease spread and possible interventions. A likelihood score is calculated for each particle describing its degree of similarity to the observed data. These scores are used to assign the particle weights which are subsequently used to re-sample the particles to produce a new particle population. An important contribution to the literature, this work however only focuses on adjusting the *parameter values*, and does not attempt to correct the model states at each time-step, so is more akin to dynamic model calibration than data assimilation.

The second study^36^ develops an ABM of COVID-19 spread that is calibrated using daily epidemiological observations and an ensemble Kalman filter. The disease model is similar to that employed here. Agents are characterised by one of seven disease states and the house and neighbourhood in which they live. At each daily model time-step the agents make contact with each other, and where a susceptible agent interacts with an infected agent, the former agent may also become infected. However, as only population-level data are available, in this case data assimilation is used to compare an aggregated count of infected agents (the ‘macro-state space’) against observations of the same, and then apply a ‘macro-micro map’ such that the individual agent states (the ‘micro-state space’) match the aggregate observations. The challenge of this approach is in disaggregating the adjusted estimate of population level disease statuses back into disease statuses for each individual^36^.

Following the work of Tabataba^30^ and Coccuci^36^ this paper applies data assimilation to the global parameters, rather than to the state space of the underlying individual-level model (i.e. by manipulating the states of the synthetic individuals directly). The manipulation of the model state space is an interesting avenue for future work^37^ but has currently only been attempted with relatively simple models with very few agents^8,10,11^. This work extends the previous work by using Approximate Bayesian Computation to update the model in response to new data. Also, the underlying disease model is extremely comprehensive, containing approximately 800k unique agents, numerous agent activities (shopping, working, schooling, etc.) and a spatially-realistic geographic environment.

## Data and methods

This paper attempts to improve the predictive accuracy of an existing individual-based disease transmission simulation^13^ (the ‘Dynamic Model for Epidemics’, DyME) through the application of a Bayesian optimisation technique (‘Approximate Bayesian Computation’) in response to weekly COVID-19 test data.

### COVID-19 data in England

The UK Government publishes the number of positive COVID-19 PCR test results over rolling 7-day windows for each middle-layer super output area (MSOA; neighbourhood areas with approximately 5,000 households). However, there are two main drawbacks to using such raw data in a disease model. First is the time delay between actually contracting the disease, developing symptoms, and taking a test. DyME simulates *disease transmission*, which will have occurred some days before a positive test is taken. Second, only a portion of all infections are identified through testing (particularly with respect to asymptomatic cases). To overcome these issues, positive case numbers per day are converted into an estimate of the number of new *infections* as follows:

1. Test data are shifted back in time by 6 days to allow for a lag between the time of a positive test result and the time of infection. This assumes that tests are always taken due to the presence of symptoms, and is based on evidence of the length of the COVID-19 incubation period^38^.
2. Monthly multipliers^39^ are applied to the data to correct for the under-reporting of cases that occurs due to: asymptomatic transmission; symptomatic people not being tested; and tests not correctly identifying all infections. These multipliers also allow for the evolution in testing capacity during the pandemic.
3. Daily values are estimated through a smoothed linear interpolation of the weekly data. This overcomes the large fluctuations in daily test numbers found in the data that are unlikely to accurately represent true transmission rates.

**Figure 1:**
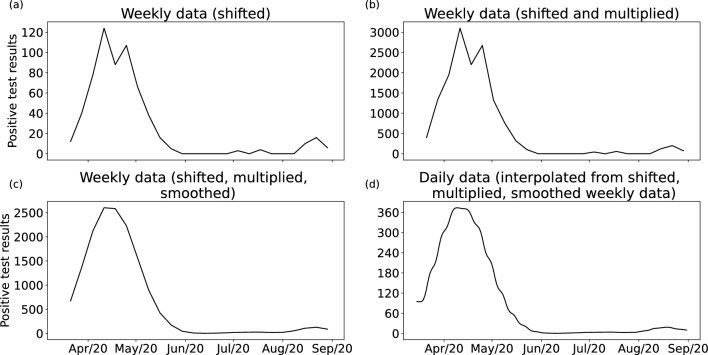
Number of positive test results across the whole of the study area with the corrections applied: (**a**) weekly positive test results are shifted back in time 6 days; (**b**) multipliers are applied to account for missed tests; (**c**) data are smoothed; (**d**) daily data are inferred by interpolating from the weekly data.

Figure 1 shows the number of cases from April to September 2020 aggregated across all the MSOAs in the study area (Devon, UK, as discussed below) at each stage of the process.

DyME creates daily estimates of neighbourhood-level COVID transmission counts. Currently these estimates are aggregated to create a single, weekly transmission estimate for the whole study area, with these aggregate estimates used to assess the model’s performance. Although daily, neighbourhood-level model validation will give a more robust estimate of the accuracy of the model and will enable an assessment of how well the model is simulating emerging micro disease clusters, estimating precise daily neighbourhood transmission counts is extremely challenging for any model. Hence we leave this level of validation for future work and use weekly estimates for the whole study (Fig. 1c) area as our validation data.

The earliest available COVID-19 test data begin on the 28th March 2020. The experiments performed for the purpose of this paper therefore begin on 16th March. Our choice of the 16th takes account of the 6 day lag period for testing, such that positive test results in the week ending the 28th March are equivalent to cases for the week ending the 22nd March. When these weekly data are interpolated to daily counts, the start of that week is the 16th March.

### Dynamic model for epidemics (DyME)

The Dynamic Model for Epidemics (DyME) is a spatial microsimulation model that combines methods from epidemiological modelling, spatial analysis & modelling, synthetic population generation and data integration^13^. The model is based on an individual-level synthetic population of all individuals in a study area, with attributes that represent individuals’ socio-economic, demographic, activity and health information. The model runs on a daily timestep, with individuals undertaking visits to shops, schools and workplaces as appropriate, as well as spending time with their household at home. Infected individuals impart a *hazard* on locations that they visit proportional to the amount of time they spend there and their degree of infectiousness. Others who visit these locations receive a degree of *exposure* which increases their chances of contracting the disease themselves. The amount of time spent at non-home locations in the model is configured to reflect actual behavioural trends; during periods with the most prohibitive rules on movements, much more time was spent at home than on other activities. When the simulation begins, an initialisation process *seeds* the first COVID-19 cases into the synthetic population by forcing the number of infections in an area to perfectly match the real observations for a period of 14 simulated days. After this period the seeding ends and the disease propagates without interference. Currently the model is applied to a case study in the region of Devon, UK, with approximately 800,000 individuals^13^.

### Approximate Bayesian computation (ABC)

Previous works^30,36^ have explored the use of particle and Kalman filters as a means of updating their individual-level epidemic models with up-to-date data. Here we attempt a similar objective but make use of a technique called Approximate Bayesian Computation (ABC). ABC is a form of Bayesian inference, initially defined and applied in the biological sciences^40–43^. It can be used to iteratively update estimates of the posterior distribution of model parameters as more evidence becomes available, but unlike other Bayesian inference techniques it does not require evaluation of the likelihood function. The likelihood function expresses the probability of obtaining some observed data given a model, but in many cases is analytically intractable or too costly to evaluate. Instead, ABC approximates the posterior distribution of the parameters by running a generative model many times with parameters drawn from a prior distribution.

ABC offers an interesting means of updating an individual-based model (IBM), specifically, for a number or reasons. Most importantly, ABC provides an assessment of the uncertainty in each parameter estimate^44^ which can in itself reveal new information about the model and the real world. For example, if we observe a model posterior parameter estimate changing over the course of a simulation run then it may tell us something about how the disease, or society’s response to it, is changing. In addition, ABC is consistent with the Pattern Oriented Modelling protocol^28^ because it iteratively explores various model ‘patterns’ that may or may not match some observation data^45^. Pattern Oriented Modelling is seen as valuable approach to ensure the robustness of empirical IBMs.

Here we briefly outline the ABC approach in general as well as the concrete implementation (sequential Monte Carlo simulation). For further detail about ABC the interested reader can refer to Turner and Zandt’s excellent tutorial^46^; we use the same notation here. In general, the question we face is how to calculate the posterior distribution, $\pi ( \theta \mid Y )$, of a set of model parameters, $\theta$, given some observed data, *Y*, using the likelihood, $L(Y \mid \theta )$, and a prior estimate of the parameters, $\pi (\theta )$:

1 $$\begin{aligned} \pi ( \theta \mid Y ) \propto L(Y \mid \theta ) \pi (\theta ) . \end{aligned}$$

In practice the likelihood function is often intractable, but recent innovations in simulation methods such as sequential Monte Carlo (SMC, also known as ‘particle filtering’^46^) and the availability of powerful computers have allowed for *approximations* of the likelihood function to be computed^46^. Here the ABC-SMC^47^ approach, as implemented using the Python package pyabc^48^ is used.

ABC works by creating a sample of candidate parameter values, $\theta ^*$, and using this to generate some simulated data, *X*. Here, DyME is used to generate *X* from the parameters $\theta ^*$ (i.e. by running the model with $\theta ^*$ as input parameter values). If the distance between the observed (*Y*) and simulated (*X*) data, termed $\rho (Y,X)$, is sufficiently small then $\theta ^*$ is retained. Here, we use the Euclidean distance between the observed number of cases per week—a vector of length 7 (one entry for each day)—and the simulated number of cases per week—an equivalent vector for the simulated number of cases in each day—as the distance function. So long as the rejection threshold is sufficiently small and the number of retained particles is sufficiently large, then the process creates an adequate approximation of the ‘true’ posterior $\pi ( \theta \mid Y )$^49^.

A problem that arises with the above approach is that the chance of a sample $\theta ^*$ generating simulated data that are sufficiently “close”^47^ to the observations is small^45^. If most samples are rejected then a large number of model runs can be required to generate a sufficiently large retained sample. This is computationally expensive. ABC-SMC tries to reduce the number of rejected samples. It operates by generating a pool of *N* candidate values for $\theta$, termed ‘particles’ (here, $N=100$). Initially the pool is created by drawing from the prior distribution, $\pi (\theta )$. The algorithm then proceeds iteratively by using the model (DyME) to generate simulated data *X* and subsequently assigning a weight, *w*, to each particle according to *p*(*Y*, *X*). Following^46^, the new weight of a given particle *i* in iteration *t* is calculated from the previous iteration ($t-1$) as follows:

2 $$w_{{i,t}} = \frac{{\pi (\theta _{{i,t}} )}}{{\sum\nolimits_{{j = 1}}^{N} {w_{{j,t - 1}} \;q\left( {\theta _{{j,t - 1}} {\mid }\theta _{{i,t}} } \right)} }}.$$

**Figure 2:**
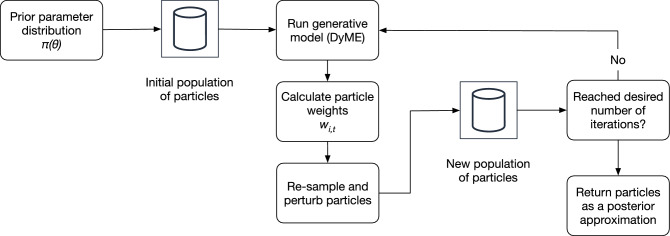
An overview of the ABC-SMC process to approximate the posterior parameter distribution.

In each iteration the particles are re-sampled and their weights re-calculated. The rejection rate should remain low because the weighting scheme is chosen to minimise the divergence between the proposed distribution and the ‘true’ posterior. Figure 2 illustrates the SMC process. For further details see^47^ or, for the full algorithm, see^46^ (Algorithm 2).

The outcome of the ABC-SMC is a population of particles, *T*, and associated weights. These particles and weights approximate the joint posterior distribution of the model parameters. Once this posterior distribution has been estimated, it is possible to use it to draw parameter values and then use the model to make forecasts. Here we first use multi-dimensional Kernel Density Estimation to create a smooth multi-dimensional surface from the particles and their weights, then draw samples of parameter values from this surface. Each sample is then used to run the DyME and make a forecast. In this way, the model forecasts retain the uncertainty associated with the posterior distributions. If ABC-SMC is able to find relatively narrow posterior estimates then the subsequent model forecasts will be similar. If, however, the parameters are less certain then the parameter samples will be quite different and the model forecasts will also produce results with a high degree of variability. The Results section demonstrates how, over the course of the experiments, the predictions become more certain as ABC-SMC is provided with more data with which to estimate the parameters.

## Experiments

The aim of the experiments performed here is twofold:

1. Evaluate the extent to which the dynamic re-calibration of an individual-level epidemic model improves the quality of its predictions of future disease transmission; and
2. Explore whether analysing the change in parameter values over time might reveal new information about the underlying dynamics of the disease, or society’s response to it.

The reminder of this section outlines the structure of the experiments that have been conducted before presenting the results in the following section.

### Parameters and priors

Table 1 outlines the initial prior distributions for each of the model parameters that are included in the dynamic calibration. The *location exposures* are parameters that influence the probability of disease transmission in different types of places. Higher values make it more likely that a susceptible person will contract the disease if the location has been visited by an infectious person. There is very limited evidence on which to estimate appropriate values for these parameters so they are assigned uniform priors. Note that the Home parameter is fixed at 1.0 such that all other transmission parameters take values that are relative to this. The *individual hazards* are parameters that determine how likely it is that a particular individual will spread the disease, given their current disease state. Previous research has pointed to the use of a Gaussian prior *N*(0.44, 0.155)^13^ which we adopt here. Note that no parameters can take values below 0 as the behaviour of the model is unpredictable in these circumstances. In the few cases where ABC sampling draws parameter values that are less than 0 these samples are discarded and new ones drawn.

**Table 1:** Prior distributions for the model parameters.

Parameter	Description	Prior
Location exposures		
Home	Exposure at home	1.0 (fixed)
Retail	Exposure associated with shopping	*U*(0, 1)
PrimarySchool	Exposure associated with primary school (children aged 4–11)	*U*(0, 1)
SecondarySchool	Exposure associated with secondary school (children aged 11–18)	*U*(0, 1)
Work	Exposure associated with attending a workplace	*U*(0, 1)
Individual hazards		
asymptomatic	Transmission hazard associated with asymptomatic individuals	*N*(0.4, 0.2)
presymptomatic	Transmission hazard associated with presymptomatic individuals	*N*(0.4, 0.2)
symptomatic	Transmission hazard associated with symptomatic individuals	*N*(0.4, 0.2)

### Dynamic re-calibration

**Figure 3:**
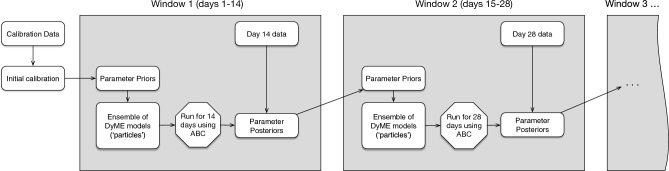
The dynamic calibration workflow.

After the initial prior parameter distributions have been specified, the dynamic re-calibration experiments begin. Figure 3 outlines the structure of the dynamic calibration workflow. Our hypothetical scenario begins on 16th March (as discussed in Data and Methods). We assume that the pandemic is currently evolving and that new infection data are emerging each fortnight, as such the process involves simulating over a ‘window’ of 14 days and re-calibrating the model at the end of each window, at which time we imagine that new data will have arrived. Hence the first window finishes on 30th March, at which point the first re-calibration begins.

ABC-SMC is used to combine the DyME model and up-to-date disease data to create an approximated posterior parameter distribution. This posterior is then used for three different purposes:

1. It forms the prior parameter distribution in the next window;
2. It is used to make predictions of the propagation of the disease over the next 63 days (9 weeks); and
3. It is stored for later analysis to see whether the change in parameter posteriors in each window might reveal useful information about the evolution of the disease and society’s response to it.

In each ABC-SMC re-calibration, 10 populations and 100 particles are used. The process is repeated each fortnight up until 22nd June (7 re-calibration windows), at which point the final prediction of 63 days (9 weeks) is made. This time period was chosen because it allows our predictions to capture three distinct periods in the disease evolution in 2020: a period of high growth of the disease when it first enters the country, a national lockdown that suppresses the disease, and then the relaxing of restrictions during the summer months.

After re-calibration, the posterior distribution from the final population in each window is then used to predict the evolution of the disease in the following 63 days (9 weeks). We posit that this prediction could be used by policy makers to plan strategy, as happened during the pandemic^2,50^. Importantly, a major advantage of ABC is that, by providing posterior parameter estimates, rather than estimates of single, ‘optimal’ parameter values, multiple model runs can be used to make predictions by drawing parameter values from the posteriors. This provides information about the *uncertainty* associated with the predictions.

Note that although the parameter posteriors are retained after each window as they are used as priors in the subsequent window, the individual states of the DyME models (the ‘particles’) are not retained. Hence new particles are created at the beginning of each window using the most recently-generated posteriors. For example, in window 3 (days 29–42) the DyME models use the posterior parameter values from window 2 (days 15–28) as their priors, but begin simulating from day 1 and run for 42 days.

## Results

This section outlines the results of the experiments. It begins by demonstrating the accuracy of the model fit achieved using ABC by comparing the data simulated from the model during the calibration period to the observed data for the same period. It then looks in more detail at the evolution of the parameter posterior distributions and finally demonstrates how well the model is able to forecast the future evolution of the disease.

### Accuracy of model fit

**Figure 4:**
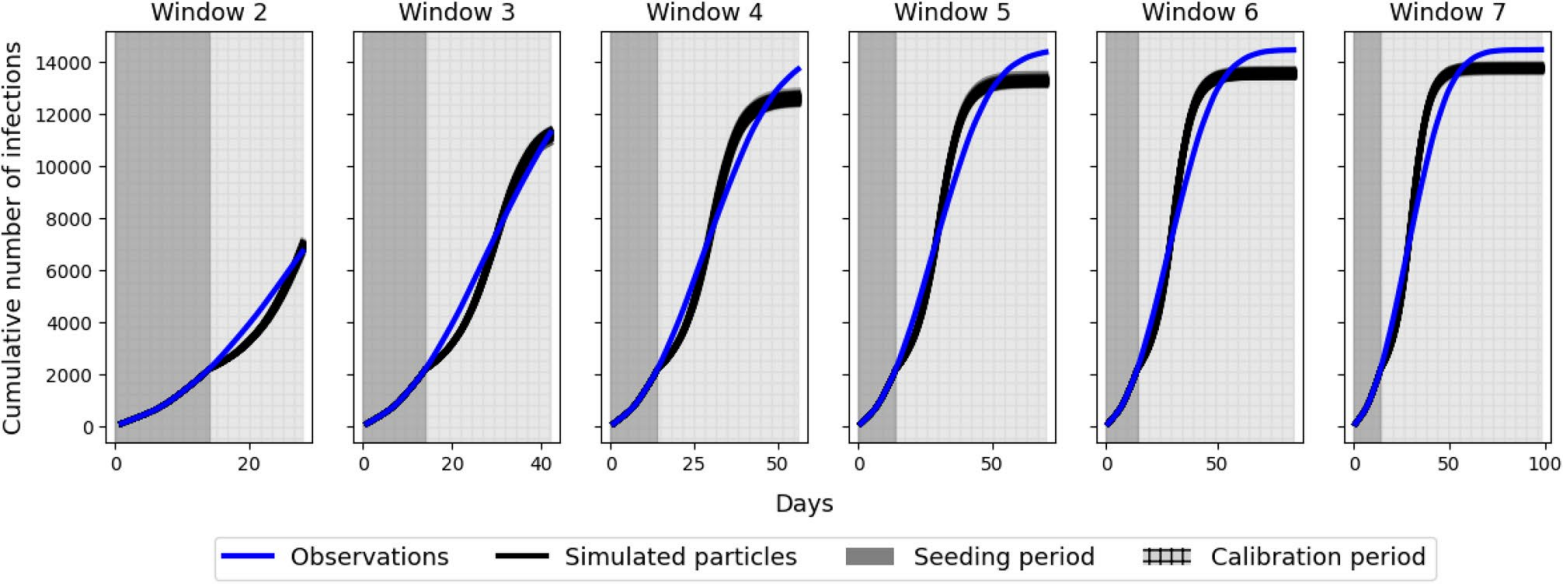
Comparing the cumulative number of infections predicted by the model (black lines; one for each particle) to those observed in the real data (blue line).

Figure 4 compares the cumulative number of infections predicted by the model in each simulated day within the calibration period to the cumulative number of infections in the observations. As there are 100 particles used in our implementation of the ABC algorithm, the ‘model prediction’ is actually made up of the 100 separate predictions made by the 100 different model instances. The ‘window’ size is 14 days, so parameter re-calibration takes place every 14 days. This was chosen because it balanced the benefits of regular re-calibration with the computational costs of doing so (the model is computationally expensive and many thousands of individual model runs are required to generate a posterior distribution for a given window with ABC-SMC). Note that the initialisation/seeding period is also 14 days, so for the first simulation window the number of infections in the observations and model predictions are identical, so only windows 2 onwards are plotted.

To supplement the visual analysis in Figure 4, we also calculate the continuous ranked probability score (CRPS) to quantify the similarity between model predictions and the observations. The CRPS is often used to evaluate the accuracy of ‘raw’ ensemble forecasts created by probabilistic models^51^. Low scores imply greater accuracy. Table 2 presents the CRPS, calculated for each window. Interestingly the score increases over time, but this is likely due to the fact that in later windows the prediction is made over a larger number of days so, all things being equal, later predictions are more likely to diverge. When we normalise by dividing the score by the number of days in the window we see the score decrease with the window number.

**Table 2:** The continuous ranked probability score (CRPS) calculated for each prediction window.

Window	CRPS	Days	Normalised CRPS
2	485	14	129
3	525	28	99
4	681	42	105
5	778	56	104
6	792	70	95
7	749	84	82

Although the model is not perfect—in particular it under-estimates the total number of infections observed in the data—it replicates the observed cumulative distribution of infections reasonably well. This imperfection is true of all of the disease spread models used during the pandemic. Importantly, the point at which the disease evolves from its rapidly expanding phase to more of a steady state (around day 60) is similar for both the model and the data. As the ‘Model Forecasts’ section will illustrate, were the model not re-calibrated dynamically then the model estimate of the number of infections quickly diverges from the real observations.

### Parameter evolution

At the end of each 14 day window we assume that new COVID-19 data become available. At this point the ABC algorithm takes the parameter distributions from the previous window as priors and then uses the model and new observations to create new, updated, posterior parameter distributions. Not only do these new posteriors allow the model to make forecasts that should be more reliable, as the newly-updated parameter distributions are able to better represent the current system state, but the distributions themselves have the potential to provide new information about the evolution of COVID-19 in a population.

**Figure 5:**
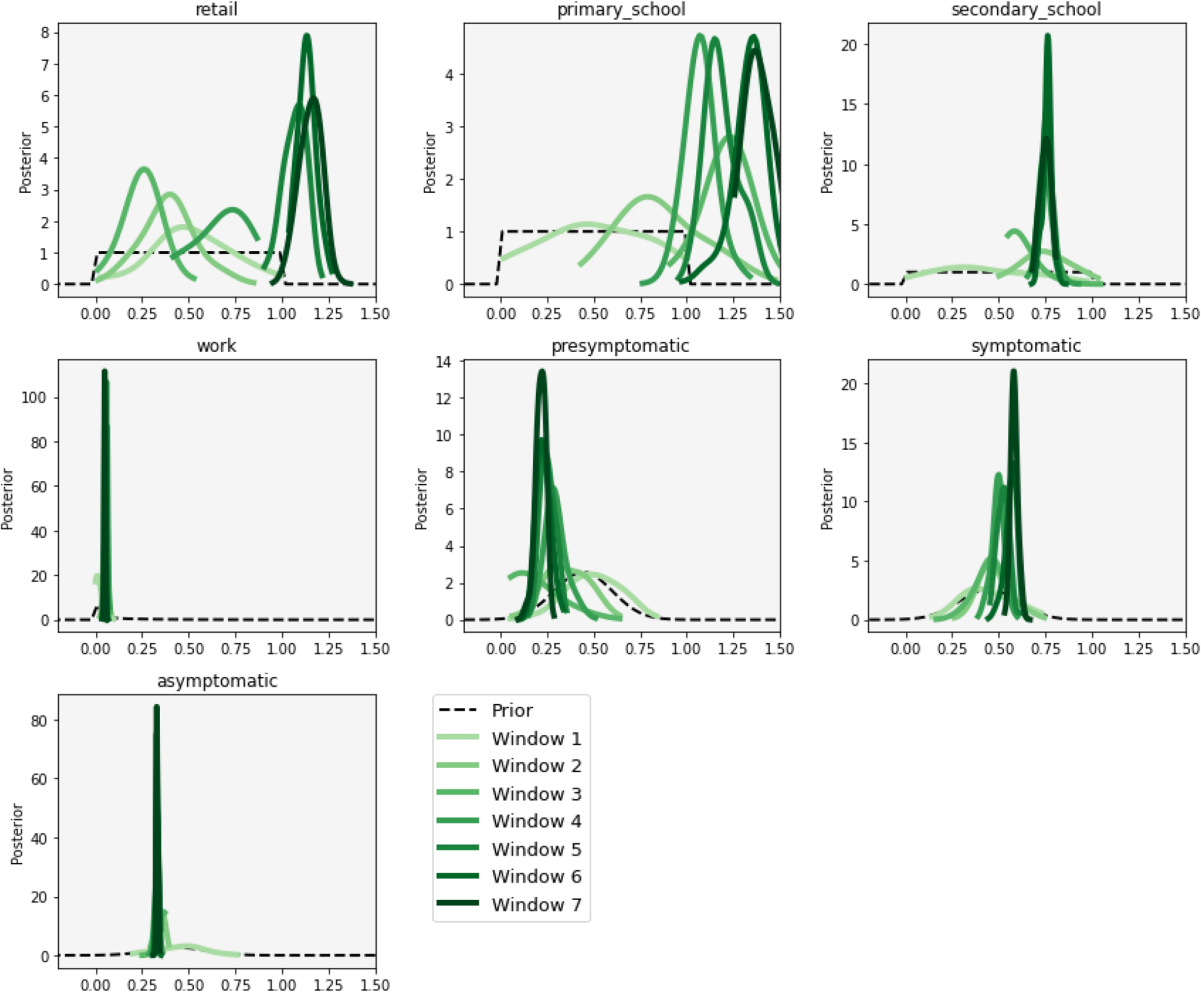
Posterior distribution of each parameter value at the end of each data calibration window, with the prior parameter distributions shown by the black dotted line.

For example, Fig. 5 presents the distributions of each parameter posterior at the end of each window. For some parameters, such as work or asymptomatic, the distribution changes little over the course of the experiment. This suggests that the exposure risks of susceptible people attending workplaces, and the individual hazards associated with asymptomatic transmission, both remained fairly consistent throughout the course of the simulation time period. Interestingly, however, the exposure risks associated with visiting retail establishments or primary schools, as well as the hazards associated with symptomatic transmission, appeared to increase in each window. There are a number of possible explanations for these findings that will be discussed in more detail in the Conclusions section.

### Model forecasts

Finally, this section illustrates the advantages that dynamic calibration offers for making more reliable forecasts of the potential future evolution of COVID-19. Recall that at the end of each calibration window a joint parameter posterior distribution is estimated. To make forecasts we draw 30 samples from this posterior (30 is sufficient to provide consistent results without being overly computationally expensive) and, for each sample of parameters, run the model for a time equal to the length of the calibration window plus 63 days (9 weeks). This length of time was chosen as it would allow for policy makers to forward plan, without forecasting too far into the future (which is extremely difficult for complex systems). For example, if we are making a forecast at the end of the second window (day 28) then we simulate for $28+63=91$ days in total.

**Figure 6:**
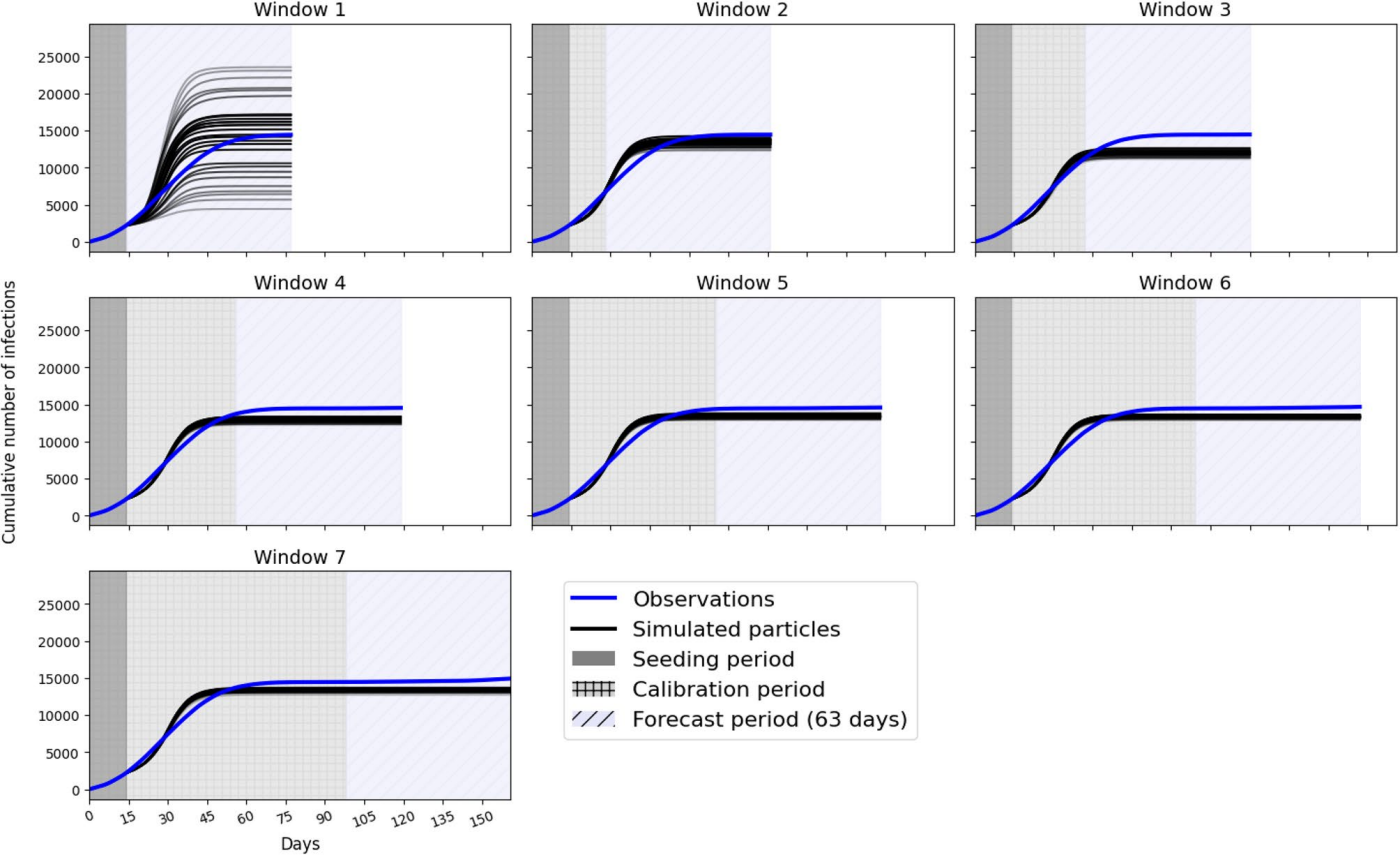
Model predictions generated by taking 30 samples from the posterior distribution at the end of each calibration window and running the DyME model for an additional 63 days.

Figure 6 plots these 30 forecasts that are conducted at the end of each window and compares them to the observations (in reality these future observations would not be available at the time). The most striking observation is that the forecasts made in early windows are much more uncertain than those in later windows. As more observational data become available the model forecasts become more certain. This has implications for the communication of model forecasts to policy makers as it becomes possible to quantify the forecast uncertainty. In addition, Table 3 illustrates the CRPS for the 63 day forecast at the end of each window. Broadly we observe a decreasing score, implying an increase in the fit of the model to the observations. Hence as a longer data time series becomes available the quality of the model forecast increases. The exception is with respect to Window 2 that exhibits an unusually low score; we discuss this finding in the Conclusions.

**Table 3:** The continuous ranked probability score (CRPS) for the 63 day forecast conducted from the end of the calibration period in each simulation window.

Window	CRPS for the forecast period
1	4119
2	1273
3	2457
4	1903
5	1266
6	1316
7	1368

## Discussion and conclusions

This paper outlines the process of using Approximate Bayesian Computation (ABC) to dynamically re-calibrate a COVID-19 model (‘DyME’) as new infection data become available. In addition, during each calibration ‘window’, samples of parameters are drawn from the posterior distribution and these are used to make forecasts of the future evolution of the disease. This is advantageous because the predictions retain the inherent parameter uncertainty that is expressed in the posterior distributions. Were a more traditional ‘one shot’ calibration method used, only a single set of ‘optimal’ parameters would be used to make forecasts. The importance of retaining this uncertainty in the parameter estimates showed itself vividly in Fig. 6. Forecasts made at the beginning of the pandemic, when only limited data were available, demonstrated extremely large uncertainty, in many cases diverging rapidly from the true evolutionary path of the disease. Such information would be crucial for policy makers who needed to make decisions based on the outcomes of the model.

With respect to the evolution of the parameter posteriors, Fig. 5 showed that some parameters change distinctively as the disease evolved. Whilst these are preliminary results and would require further exploration before drawing any firm conclusions, two interesting outcomes can be noted with respect to *location exposures* and *individual hazards*. With respect to exposure, we see that whilst the risks of attending workplaces and secondary schools remained fairly constant, the risks of contracting the disease from an exposed person in shops and primary schools increased over the course of the simulation. This may be because workplaces generally implemented strict disease control measures, or perhaps because workplaces remained closed for a longer period of time than shops and schools (although this does not explain why the location exposures related to secondary schools remained constant). With respect to individual hazards, it appears that the risk of infection from symptomatic people increased. This could be due to a societal response to the disease—e.g. over time people begin to behave more normally even though they have symptoms – although, as with schools, this would not explain why the hazard associated with presymptomatic transmissions appeared to decrease. Ultimately, the results show that there is value in producing dynamic parameter posteriors as these might reveal useful information about the propagation of the disease, and society’s evolving reaction to it, even though at this stage it is not possible to draw any firm conclusions regarding the specific dynamics.

A related problem is that we are trying to draw conclusions from the complex evolution of a simulation that is underpinned by limited data. Even with the regular addition of new infection data, there are still large parts of the system for which no data are available. The DyME implements a rich model of the daily behaviour of the $\sim$800,000 synthetic individuals in the study area, whereby disease spread occurs as individuals commute, shop, and attend schools. However, there are very limited data that can be used to firmly quantify the impact of these activities on the spread of a disease. This results in the situation where the model of the system is extremely rich and detailed, but the associated data are extremely sparse. Whilst it is possible to gather some additional data, it will always be the case with individual-level models such as DyME that the model is richer than the available data. Hence the use of parameter inference such as that demonstrated here might help to shed light on the true underlying dynamics in the absence of real data.

The difficulty in fully validating all model components, which is related to the overall complexity of the model, goes some way to explain the unexpectedly high forecast accuracy in Window 2 (see Table 3). We suspect that this result relates to the (in)ability of the model to simulate different stages of the evolution of the disease with one set of parameter values, and the modelling set-up which requires the model simulations to always be restarted from Day 1 (as explained in the ‘Dynamic Recalibration’ section). We see in Fig. 6 that in Window 2, ABC estimates parameter posteriors that capture the rapid spread of the disease in the early stages of the pandemic. When these posteriors are used to simulate forward, it becomes apparent that although these parameter estimates cause the number of infections in the model forecasts to eventually stabilise at a level very similar to the true infection count in the observations, the initial rise in infections is too rapid and the models diverge from the observations at the start of the Window 2 forecast ($\sim$ days 28–42). In Window 3, ABC includes the observations for this period (days 28–42) in its calibration and thus attempts to find parameters that fit the rise in infections more accurately. We see in Fig. 6 that although this is achieved, the chosen parameter combinations subsequently cause infection rates to level out earlier and at lower values than in the observations (and, importantly, with less accuracy than is seen in Window 2). This implies that if it was possible to avoid restarting the model from Day 1 each time then its likely that the dynamic re-calibration with ABC would result in a more pronounced improvement in model accuracy. This surprising finding highlights an advantage of the re-application of ABC in different windows; future work should revisit the model to better understand the dynamics of the initial rise in infections and why, if this rise is simulated accurately, the model under predicts the total infection count.

Although dynamic re-calibration is shown to improve model predictions, the overall aim of this programme of work is to implement *data assimilation* algorithms that are able to update the model *state* as well as its parameters in light of new data. This approach may be even more successful because it would allow manipulation of the state of individual model instances. For example, it may be that a cluster of cases begins to emerge in a particular neighbourhood, but the only way to ‘nudge’ the model towards such a scenario would be to directly manipulate its state by changing the infection status of the individuals in that neighbourhood. Whilst this is an exciting possibility for individual-level disease modelling, the field needs much more work in adapting data assimilation methods for large, complex individual-level models first. Currently the only published examples of the use of data assimilation in the context of individual-level modelling are on relatively simple systems^8–12^.

In summary, this paper has presented an approach to the dynamic re-calibration of an individual-level model of disease spread. Although the research is still in early stages, the results are promising and immediate next steps will be to explore whether full data assimilation will be feasible for such a large and complex individual-level model.

## Data Availability

There are two sources of data used in this study. COVID-19 test data. As discussed in the ‘Data and Methods’ section, aggregate positive COVID-19 test results are derived from publicly-available data published by the UK Government (https://coronavirus.data.gov.uk/details/download). The derived data used in the paper are stored in the code repository, archived with DOI 10.5281/zenodo.7303655. Synthetic population data. The Dynamic Model for Epidemics (DYME)^13^ uses synthetic population data to derive the simulated population of households and individuals. These data are downloaded automatically when the model is executed for the first time. They have also been permanently archived using the Open Science Foundation with DOI 10.17605/OSF.IO/QZW6F All data used in the project are synthetic and anonymous. No real people or households can be identified.
